# Advanced Restriction Imaging and Reconstruction Technology for Prostate Magnetic Resonance Imaging (ART-Pro): A Study Protocol for a Multicenter, Multinational Trial Evaluating Biparametric Magnetic Resonance Imaging and Advanced, Quantitative Diffusion Magnetic Resonance Imaging for the Detection of Prostate Cancer

**DOI:** 10.1016/j.euros.2024.12.003

**Published:** 2024-12-20

**Authors:** Madison T. Baxter, Christopher C. Conlin, Aditya Bagrodia, Tristan Barrett, Hauke Bartsch, Anja Brau, Matthew Cooperberg, Anders M. Dale, Arnaud Guidon, Michael E. Hahn, Mukesh G. Harisinghani, Juan F. Javier-DesLoges, Sophia C. Kamran, Christopher J. Kane, Joshua M. Kuperman, Daniel J.A. Margolis, Paul M. Murphy, Nabih Nakrour, Michael A. Ohliger, Rebecca Rakow-Penner, Ahmed Shabaik, Jeffry P. Simko, Clare M. Tempany, Natasha Wehrli, Sean A. Woolen, Jingjing Zou, Tyler M. Seibert

**Affiliations:** a Department of Radiation Medicine and Applied Sciences, University of California San Diego School of Medicine, La Jolla, CA, USA; b Department of Radiology, University of California San Diego School of Medicine, La Jolla, CA, USA; c Department of Urology, University of California San Diego School of Medicine, La Jolla, CA, USA; d Department of Radiology, Addenbrooke’s Hospital, Cambridge University Hospitals NHS Foundation Trust, Cambridge, UK; e GE Healthcare, Chicago, IL, USA; f Department of Urology, University of California San Francisco, San Francisco, CA, USA; g Department of Epidemiology & Biostatistics, University of California San Francisco, San Francisco, CA, USA; h Department of Neurosciences, University of California San Diego School of Medicine, La Jolla, CA, USA; i Halıcıoğlu Data Science Institute, University of California San Diego, La Jolla, CA, USA; j Department of Radiology, Massachusetts General Hospital, Boston, MA, USA; k Department of Radiation Oncology, Massachusetts General Hospital, Boston, MA, USA; l Department of Radiology, Weill Cornell Medical College, New York, NY, USA; m Department of Radiology and Biomedical Imaging, University of California San Francisco, San Francisco, CA, USA; n Department of Pathology, UC San Diego School of Medicine, La Jolla, CA, USA; o Department of Pathology, University of California San Francisco, San Francisco, CA, USA; p Department of Biostatistics, Herbert Wertheim School of Public Health & Human Longevity Science, University of California San Diego, La Jolla, CA, USA; q Department of Bioengineering, University of California San Diego Jacobs School of Engineering, La Jolla, CA, USA

**Keywords:** Biparametric magnetic resonance imaging, Clinical trial, Multiparametric magnetic resonance imaging, Prostate cancer, Restriction Spectrum Imaging, Restriction Spectrum Imaging restriction score

## Abstract

Multiparametric magnetic resonance imaging (mpMRI) is strongly recommended by current clinical guidelines for improved detection of clinically significant prostate cancer (csPCa). However, the major limitations are the need for intravenous (IV) contrast and dependence on reader expertise. Efforts to address these issues include use of biparametric magnetic resonance imaging (bpMRI) and advanced, quantitative magnetic resonance imaging (MRI) techniques. One such advanced technique is the Restriction Spectrum Imaging restriction score (RSIrs), an imaging biomarker that has been shown to improve quantitative accuracy of patient-level csPCa detection. Advanced Restriction imaging and reconstruction Technology for Prostate MRI (ART-Pro) is a multisite, multinational trial that aims to evaluate whether IV contrast can be avoided in the setting of standardized, state-of-the-art image acquisition, with or without addition of RSIrs. Additionally, RSIrs will be evaluated as a stand-alone, quantitative, objective biomarker. ART-Pro will be conducted in two stages and will include a total of 500 patients referred for multiparametric prostate MRI with a clinical suspicion of prostate cancer at the participating sites. ART-Pro-1 will evaluate bpMRI, mpMRI, and RSIrs on the accuracy of expert radiologists’ detection of csPCa and will evaluate RSIrs as a stand-alone, quantitative, objective biomarker. ART-Pro-2 will evaluate the same MRI techniques on the accuracy of nonexpert radiologists’ detection of csPCa, and findings will be evaluated against the expertly created dataset from ART-Pro-1. The primary endpoint is to evaluate whether bpMRI is noninferior to mpMRI among expert (ART-Pro-1) and nonexpert (ART-Pro-2) radiologists for the detection of grade group ≥2 csPCa. This trial is registered in the US National Library of Medicine Trial Registry (NCT number: NCT06579417) at ClinicalTrials.gov. Patient accrual at the first site (UC San Diego) began in December 2023. Initial results are anticipated by the end of 2026.

## Take Home Message

ART-Pro is a multicenter, multinational trial evaluating advanced magnetic resonance imaging techniques for improved prostate cancer detection. The study will be conducted in two phases and will evaluate whether modern technologies can overcome the need for intravenous contrast and dependence on reader expertise.

## 1 Introduction and hypotheses

Clinical guidelines strongly recommend multiparametric magnetic resonance imaging (mpMRI) prior to biopsy to improve the detection of clinically significant prostate cancer (csPCa) [1], [2]. Multiparametric MRI helps avoid unnecessary biopsies and improve the detection of csPCa through the use of MRI-targeted biopsy, compared with systematic biopsy alone [1], [2], [3], [4]. With prostate cancer (PCa) diagnoses currently at 1.4 million per year in 2020 and expected to double by 2040—and considering that millions more men will be evaluated for possible cancer—there is a critical need to dramatically increase the capacity for prostate MRI [5]. Access to prostate MRI is already limited, creating a health disparity that often disproportionately affects those at the highest risk of dying from PCa [6], [7]. The major limitations to scaling up capacity for mpMRI prior to biopsy are dependence on reader expertise and the need for intravenous (IV) contrast.

Prostate mpMRI interpretation is dependent on reader expertise and inherently subjective. Despite guidelines to standardize image acquisition and reporting (Prostate Imaging Reporting and Data System [PI-RADS]) [8], results vary widely between radiologists and imaging centers [9], [10]. To achieve good results with mpMRI, radiologists must gain significant training and experience [11], specifically for prostate MRI [12], which often requires years and considerable resources. A rapid increase in the supply of expert prostate radiologists may not be feasible, and while artificial intelligence (AI) will very likely help fill this void, development of such tools will require large, well-annotated, standardized patient cohorts to develop and validate such AI-based tools. Changes in imaging technology (eg, scanners, operating systems, and/or reconstruction methods) may also have unpredictable effects on deep learning AI models. Imaging quality is also variable, a reflection on both the heterogeneity of MRI equipment and the experience of imaging center staff in designing and following PCa acquisition protocols. Although PI-RADS lists some technical specifications, there is still a nearly unlimited range of permissible protocols for acquiring prostate MRI data, even on the same scanner [8]. Vendor, scanner model, and operating system versions add further opportunity for complexity. Additionally, many prostate MRI scans are not compliant with the minimal PI-RADS technical specifications. Two multisite studies evaluating adherence to individual PI-RADS acquisition parameters found that fewer than 20% of scans were compliant [13], [14]. Further standardization of prostate MRI protocols beyond meeting the minimal PI-RADS specifications, for example, using Prostate Imaging Quality (PI-QUAL) [15], has the potential to greatly improve consistency of images.

Biparametric MRI (bpMRI) is mpMRI without IV contrast. Biparametric MRI avoids the issues associated with invasive gadolinium contrast injection, including increased patient risk, limited accessibility, and increased time and cost. First, obtaining IV access requires a skilled technologist or nurse, and because administration of contrast presents risks to patients, in the USA, physicians are required to be present during administration to monitor for potential adverse effects, placing a constraint on the ability of many centers to meet the high demand for scans in the setting of decreased physician availability and increased labor costs. These limitations may be even more pronounced in remote or underserved areas. Additional cost considerations associated with mpMRI include the contrast agent itself and the increased scan time (and reduced scanner availability) due to the dynamic contrast-enhanced (DCE) sequence [16], [17]. Finally, while the clinical relevance is not fully understood, literature suggests that there are potential health impacts of gadolinium contrast exposure, including gadolinium deposits in the brain and other parts of the body [18], [19]. Thus, alleviating the need for IV contrast by the use of bpMRI affords the advantages of improving patient comfort, increasing capacity, saving time, and reducing cost. There is evidence that bpMRI may be comparable with mpMRI for guiding biopsy decisions [20], although results are mixed. First, when using bpMRI only, radiologists tend to find more false-positive lesions, leading to unnecessary biopsies [21]. Second, DCE helps nonexpert radiologists detect suspicious regions that they could otherwise miss with bpMRI, resulting in overall higher sensitivity than that of bpMRI alone [22]. Third, DCE also often serves as a backup when diffusion-weighted imaging (DWI) is of poor quality [21], [23], so as to avoid the use of contrast, DWI needs to be of high quality consistently. A prospective international study (PRIME) comparing bpMRI and mpMRI has fully accrued, with preliminary results presented at the 2024 European Association of Urology (EAU) meeting showing comparable outcomes with the two approaches [24]. PRIME included a lead-in period for each participating site to evaluate and ensure high quality. Overall, bpMRI may potentially remove one barrier (IV contrast) to increase prostate MRI capacity while exacerbating another barrier (dependence on examination quality and radiologist expertise).

Prostate MRI quality may benefit greatly from new MR technological advances. Scanner hardware continues to improve, including addition of surface coils that can be placed on the patient to yield higher signal to noise. Modern scanners with higher field strength (3.0 Tesla) do not require an endorectal coil to be inserted into the patient. Higher gradient performance yields better DWI, a critical part of bpMRI and mpMRI. Software advances, too, can make important contributions to image quality. Reconstruction techniques that leverage deep learning AI enable rapid acquisition of high-quality images [25]. Another approach to mitigate image quality issues is to standardize acquisition protocols for prostate MRI across centers, at least for a given vendor and software version.

Restriction Spectrum Imaging (RSI) is an advanced diffusion technique that can generate images with high specificity for csPCa [26], [27], [28], [29]. RSI can be performed efficiently on clinical scanners using standard pulse sequences at multiple *b* values (diffusion weightings) to distinguish signal from four discrete tissue microcompartments (intracellular water, extracellular hindered water, freely diffusing water, and flowing fluid) [26], [27], [28], [29]. Retrospective studies have shown the potential for RSI to make csPCa more visible and to improve radiologist accuracy [30], [31]. RSI also lends itself to superior correction of DWI distortion (eg, from rectal gas) that can interfere with MRI quality (and may be even more important for studies performed without contrast sequences as an image quality “safety net”) [26], [32], [33]. Beyond subjective interpretation, RSI yields a quantitative imaging biomarker, the RSI restriction score (RSIrs), that is superior to conventional apparent diffusion coefficient (ADC) for patient-level detection of csPCa [34], [35]. The maximum RSIrs in the prostate can be determined automatically, without the need for a radiologist to first subjectively define a lesion of interest, and has been shown to perform similarly to expert PI-RADS interpretation for patient-level detection of csPCa [34]. These results have been replicated in a large study from the Quantitative Prostate Imaging Consortium, involving RSI data from 17 scanners and seven imaging centers [35]. A prospective study also showed that nonradiologists were significantly more likely to identify expert-defined csPCa on MRI correctly when they were given RSIrs maps, than when they used conventional mpMRI [36], [37]. As an objective biomarker, RSIrs could level the radiology playing field and facilitate more consistent interpretation of prostate MRI.

The objective of the Advanced Restriction imaging and reconstruction Technology for Prostate MRI (ART-Pro) study is to evaluate whether modern technologies can overcome two major barriers to widespread accurate prostate MRI: need for IV contrast and dependence on reader expertise. ART-Pro will be conducted in two stages. In ART-Pro-1, we will test whether IV contrast can be avoided in the setting of standardized, state-of-the-art image acquisition, with or without addition of RSIrs. This is a multisite study, and in contrast to most prior and ongoing studies, we seek to minimize variability of image quality by standardizing MRI acquisitions across all sites. We will also evaluate RSIrs as a stand-alone, quantitative, objective biomarker for the detection of csPCa. In ART-Pro-2, we will measure the impact of RSIrs and IV contrast on the accuracy of *nonexpert radiologists’* detection of csPCa. While ART-Pro-1 involves a select group of expert prostate radiologists at centers of excellence (to establish a reference standard with high-quality prostate MRI interpretation), ART-Pro-2 is a preplanned retrospective study of nonexpert radiologists’ interpretations of ART-Pro-1 images.

## 2 Design

### 2.1 Study design overview

The ART-Pro study aims to evaluate prostate MRI techniques in two stages. ART-Pro-1 is a multisite, multinational, paired cohort trial evaluating whether IV contrast can be avoided in the setting of standardized, state-of-the-art image acquisition, with or without addition of RSIrs. RSIrs will also be evaluated as a stand-alone, quantitative, objective biomarker. ART-Pro-2 is a preplanned retrospective, multisite, multinational study that leverages the (state-of-the-art, standardized) ART-Pro-1 dataset to study the impact of RSIrs and IV contrast on the accuracy of nonexpert radiologists’ detection of csPCa. The trial is registered in the US National Library of Medicine Trial Registry (NCT number: NCT06579417). The expected trial timeline is 3 yr to complete accrual, with a 6-mo endpoint.

### 2.2 Objectives

The primary and secondary objectives are listed in Table 1.

**Table 1:** Study objectives

*Primary objectives*
1. Evaluate if bpMRI is noninferior to mpMRI among expert (ART-Pro-1) and nonexpert (ART-Pro-2) radiologists for the detection of GG ≥2 csPCa
*Secondary objectives*
1. Evaluate if RSIrs plus axial *T_2_*-weighted MRI is noninferior to mpMRI among expert and nonexpert radiologists for the detection of GG ≥2 csPCa a
2. Compare quantitative RSIrs (objective interpretation) with qualitative mpMRI (subjective radiologist interpretation) for the detection of GG ≥2 csPCa a
3. Evaluate bpMRI, mpMRI, and RSIrs for avoidance of unnecessary biopsies (ie, any biopsy resulting in no cancer or only GG 1) a
4. Evaluate bpMRI, mpMRI, and RSIrs for the detection of GG ≥3 cancer
5. Assess performance of the above MRI techniques for the detection of each: GG 1 (overdiagnosis), GG 2, GG 3, and GG 4–5
6. Evaluate the quality of scan images: a. Percentage of cases with diagnostic quality imaging as per PI-QUAL b. Percentage of cases with moderate or severe distortion i. Percentage of cases where distortion correction is useful
7. Evaluate csPCa detection by targeted vs systematic biopsy cores
8. Measure inter-reader reliability of bpMRI and mpMRI
9. Evaluate accuracy of radiologists’ and RSIrs-based estimates of overall probability of csPCa on biopsy
10. Assess influence of RSIrs on bpMRI interpretation

bpMRI = biparametric MRI; csPCa = clinically significant prostate cancer; GG = grade group; mpMRI = multiparametric MRI; MRI = magnetic resonance imaging; PI-QUAL = Prostate Imaging Quality; RSIrs = Restriction Spectrum Imaging restriction score.

a Key secondary objectives.

### 2.3 Study population

We will acquire subject data from five sites: University of California San Diego (UCSD), University of California San Francisco (UCSF), Massachusetts General Brigham Hospital (MGB), Weill Cornell Medical College (Cornell), and the University of Cambridge, UK (Cambridge). Patients referred for prostate mpMRI at any of the five participating sites, with a clinical suspicion of PCa, and those who meet all the inclusion/exclusion criteria in Table 2 will be eligible for the trial. As a pragmatic study, eligibility criteria are broad to reflect the patient population currently referred for prostate MRI. We will include a total of 500 patients in the trial (100 from each of the five sites).

**Table 2:** Eligibility criteria

*Inclusion criteria*
1. 18 yr of age or older
2. Referred for mpMRI of the prostate for suspicion of prostate cancer
3. MRI is conducted using the standardized ART-Pro acquisition protocol
*Exclusion criteria*
1. Currently incarcerated
2. Previous diagnosis of prostate cancer
3. Active nonprostate tumor(s) in structures of the body near the prostate
4. Previous prostate surgery
5. History of hip implant
6. Metal implants or implanted devices in the body or other criteria that are deemed to require deviation from the usual acquisition protocol or scanning procedures

mpMRI = multiparametric MRI; MRI = magnetic resonance imaging.

### 2.4 Patient recruitment

Patients are referred for prostate mpMRI for a suspicion of PCa as per clinical routine. These patients are screened for study eligibility, and eligible patients are included in the study. The total scanner time for the ART-Pro study is comparable with that of routine clinical prostate MRI examinations, and having two expert radiologists independently and collectively interpret the images would not be expected to adversely affect clinical care. The only real risk to patients enrolled in the study is loss of confidentiality, and steps to mitigate this risk are outlined in the research protocol approved by each site’s local institutional review board. Hence, at three of the four US sites, local institutional review boards determined this study as HIPAA compliant and could be conducted with a waiver of consent, based on minimal risk to patients. This recruitment approach also ensures that patients included will be representative of the populations served by these institutions. The local institutional review board at MGB has approved patients to be enrolled with verbal consent. At Cambridge, approval has been secured by the appropriate research ethics committee to consent participants for prostate MRI with additional sequences and for sharing data outside the UK. This study was approved at UCSD on September 19, 2023; UCSF on February 4, 2024; Cornell on March 19, 2024; and MGB on July 12, 2024. Participants at the Cambridge are consented under a broader protocol for prostate MRI with additional research sequences, approved on February 18, 2020 (see the Supplementary material).

## 3 Protocol overview

### 3.1 Study schema for ART-Pro-1

**Fig. 1:**
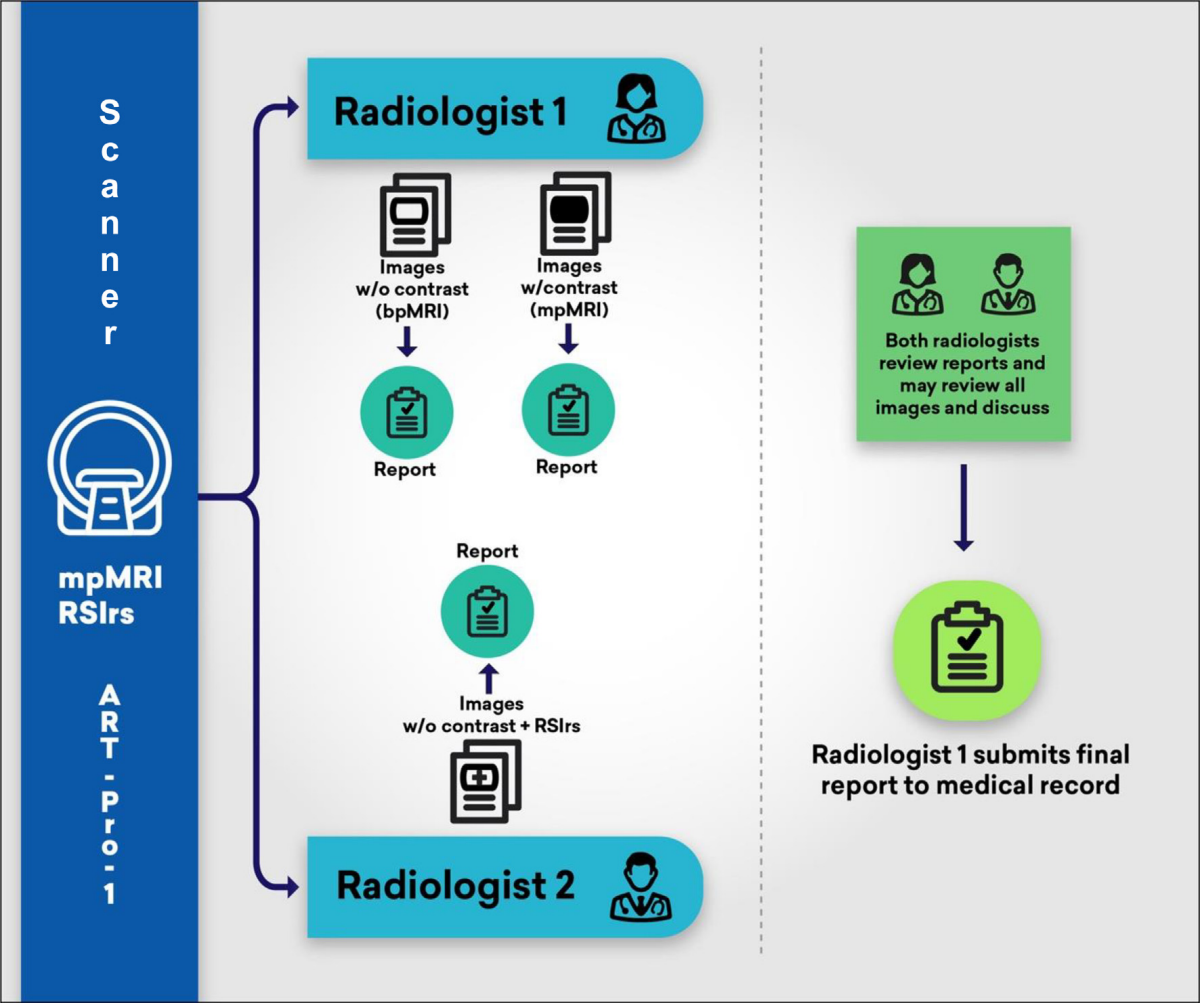
In ART-Pro-1, patients with suspected clinically significant prostate cancer (csPCa) undergo MRI, with images interpreted independently by two expert radiologists. Radiologist 1 provides a research report using only biparametric MRI (bpMRI; no contrast) and then a second research report after reviewing the dynamic contrast-enhanced images (full multiparametric MRI [mpMRI]). Radiologist 2 provides a research report using bpMRI plus Restriction Spectrum Imaging restriction score (RSIrs). After both radiologists have submitted their research reports, they are given each other’s results. They may review all images and discuss the case, if useful. Radiologist 1 submits a final clinical report to the patient’s medical record to guide biopsy decisions. MRI = magnetic resonance imaging; w/ = with; w/o = without.

The study schema for ART-Pro-1 is presented in detail in Figure 1.

### 3.2 MRI acquisition and technique

Eligible patients undergo mpMRI examination as per clinical standard of care, with additional images acquired for RSI. All examinations, in accordance with the guidelines for standard mpMRI, include T_2_-weighted (T2W) sequences, DWI sequences, and a DCE sequence. At the four US centers, RSI images are also acquired for clinical use. For ART-Pro, an additional RSI sequence (with longer echo time [TE]) is acquired for the ART-Pro study, adding 3 min and 25 s to the MRI examination. At the UK site, the three RSI sequences (approximately 10 min) are for research and are performed after obtaining written consent. Acquisitions at four of the five sites will all be performed on the same 3 T platform (SIGNA Premier XT; GE HealthCare, Waukesha, WI, USA) equipped with high-performance diffusion gradients, blanket surface coils (AIR Coils), and product deep learning–based denoising technology for T_2_ and DWI (AIR Recon DL). Acquisitions at one of the five sites will be performed on a SIGNA Architect XT equipped with the same software and hardware except using slightly lower performance gradients.

The MRI acquisition protocol for ART-Pro was designed by consensus among the investigators, including physicists, engineers, and ten genitourinary radiologists from the five participating institutions, and with support from the scanner manufacturer. Development was informed by several empirical tests and votes for consensus (see the Supplementary material). Table 3 shows the imaging parameters in detail.

**Table 3:** MRI parameters

	Acquisition time (min:s)	*b* values (s/mm^2^) [number of samples]	FOV (mm)	Matrix	Slices	Slice thickness (mm)	TR (ms)	TE (ms)
*Localizer*								
3-plane localizer	0:18	NA	300 × 300	320 × 160	NA	8	Minimum	80
*T_2_ weighted*								
Axial *T_2_*	2:07	NA	160 × 160	360 × 224	32	3	3344	102
Sagittal *T_2_*	1:44	NA	160 × 160	360 × 224	26	3	2717	102
Coronal *T_2_*	1:44	NA	160 × 160	360 × 224	26	3	2717	102
*T_1_ weighted*								
Axial *T_1_*	0:47	NA	200 × 200	256 × 256	1	3	5	2
*Conventional DWI*								
Axial reduced FOV (FOCUS)	1:53	50 [6], 1000 [18] (synth a 1400 2000)	160 × 88	100 × 50	32	3	4500	Minimum
Axial extended FOV	3:02	50 [6], 1000 [24] (synth a 1400 2000)	320 × 320	128 × 128	45	3	5088	Minimum
*RSI DWI*								
RSI with short TE	3:25	0 [5], 100 [6], 800 [12], 1400 [12], 2500 [18]	160 × 160	64 × 64	32	3	3800	80
RSI with short TE and reverse phase encoding polarity	3:25	0 [5], 100 [6], 800 [12], 1400 [12], 2500 [18]	160 × 160	64 × 64	32	3	3800	80
RSI with long TE	3:25	0 [5], 100 [6], 800 [12], 1400 [12], 2500 [18]	160 × 160	64 × 64	32	3	3800	100
*Dynamic contrast enhanced*								
Axial DISCO b	2:53	NA	160 × 160	100 × 100	1	3	2.5	Minimum
Total scan time		24:43						

DISCO = DIfferential Subsampling with Cartesian Ordering; DWI = diffusion-weighted imaging; FOCUS = Field-of-view Optimized and Constrained Undistorted Single-shot; FOV = field of view; MRI = magnetic resonance imaging; RT = repetition time; RSI = Restriction Spectrum Imaging; TE = echo time.

a Synthetic *b*-value images, automatically computed from the acquired *b*-value data using built-in vendor software.

b Additional parameters include inversion time of 23 ms, 38 phases, and 32 wash-in phases.

Three RSI scans are included in the protocol for ART-Pro: two with a short TE of 80 ms (the minimum achievable TE on the scanner hardware selected for the study) and one with a longer TE of 100 ms. The two short TE scans are acquired clinically, with opposite phase-encoding polarity (but are otherwise identical) to allow for effective correction of image distortions caused by inhomogeneities in the main magnetic field (*B_0_*) [32]. The RSIrs maps shown to radiologists during the study are the average of the RSIrs maps generated from the two opposite-polarity short TE scans. The long TE scan is included for research and is not initially reviewed by any radiologist but is included to enable future investigations into the effects of T_2_ weighting on RSI signal properties and PCa detection.

### 3.3 Innovation

Beyond standardization, the state-of-the-art MRI acquisition protocol used in ART-Pro incorporates several technological innovations. All T2W and diffusion-weighted images in ART-Pro use high-density blanket surface coils and AI-based reconstruction to improve image quality and consistency. In addition to conventional DWI, ART-Pro includes multi-*b*-value DWI to permit calculation of RSIrs maps. The novel distortion correction method based on multi-*b*-value acquisition that is applied to RSIrs maps is used to improve cancer detection in cases where rectal gas leads to compression or stretching of prostate tissue on DWI [32].

### 3.4 Image distribution and processing

Images from within each institution’s health IT network are transmitted from their scanner(s) to a processing/routing system created for ART-Pro. This system creates and processes two subsets of images, one for reader 1 and one for reader 2. The subset for reader 1 includes the standard of care mpMRI sequences (DWI, T2W, and DCE). These images are fully identifiable with the patient ID and are no different from standard of care images when sent to PACS. The subset for reader 2 includes bpMRI (without DCE) and RSIrs; these are deidentified and assigned an anonymized ID before being sent back to PACS. This deidentified work list for reader 2 ensures that reader 2 does not inadvertently view the full set of mpMRI images and/or interfere with reader 1’s clinical work list essential for clinical reporting. The processing system generates RSIrs maps using the internal MATLAB code developed by the investigator team and described previously [27], [28], [34], [35]. Briefly, the multidirection (tensor), multi-*b*-value diffusion-weighted images are corrected for image distortions arising from *B_0_*-field inhomogeneity, gradient nonlinearity, and eddy currents. Background noise and receiver coil bias are then removed. The corrected data are fit to a multicompartment RSI model, and the signal from the slowest diffusion compartment is normalized by the median signal within the prostate on the *b* = 0 s/mm^2^ images to generate RSIrs maps. RSIrs maps from both short TE scans are averaged together to generate the final RSIrs map that is distributed to the radiologists.

### 3.5 MRI interpretation in ART-Pro-1

MRI examinations are evaluated by two expert radiologists at the imaging center, with each radiologist interpreting approximately half of the cases in the role of reader 1 and half in the role of reader 2 (either by random assignment or by which radiologist happens to be on clinical service the day the patient is scanned). Reporting of MRI examinations is done as the per PI-RADS v2.1 guidelines. Both readers also assess the quality of each modality reviewed (T2W, DWI, and DCE). Both readers know the clinical indication for the prostate MRI examination given by the ordering physician. Both also have access to the patient’s electronic medical record for age, race, ethnicity, family history, prostate-specific antigen level, prior biopsy information, etc. Both readers record whatever clinical information they reviewed by copying it into their research reports in our centralized REDCap database.

#### 3.5.1 Reader 1

Reader 1 fills out a research report (Reader 1 Report; see the Supplementary material), captured and stored in our centralized REDCap database, of the MRI findings. Reader 1 is first blinded to the DCE sequence and reports the MRI using only the biparametric (T2W and DWI) sequences. After reporting the bpMRI, reader 1 is unblinded to the DCE sequence and re-reports the MRI using the full mpMRI (T2W, DWI, and DCE) sequences. Reader 1 cannot go back and change his/her response for own bpMRI findings, so reader 1 ultimately provides two research reads: one without DCE and one with DCE. For both the bpMRI and mpMRI reports, reader 1 provides an overall estimate of the probability of csPCa per lesion and per patient.

#### 3.5.2 Reader 2

While blinded to the findings of reader 1, reader 2 completes a separate research report using bpMRI and RSIrs (Reader 2 Report; see the Supplementary material), captured and stored in our centralized REDCap database. Lesions are assessed according to PI-RADS v2.1 with bpMRI; additionally, reader 2 reports the maximum RSIrs for each lesion. Reader 2 also provides an overall estimate of the probability of csPCa per lesion and per patient.

As described above, RSIrs images in ART-Pro are acquired in two opposite phase encoding directions to permit multi-*b*-value correction of distortion due to *B_0_* field inhomogeneity. Both readers are asked to indicate whether DWI is distorted significantly in each examination. If there is significant distortion in standard DWI, reader 2 also reports whether the distortion is meaningfully reduced in distortion-corrected RSI.

#### 3.5.3 Re-evaluation

Once both readers have completed their separate research reports, reader 2’s report is delivered to reader 1, the clinical radiologist of record. Upon review of reader 2’s report and after any warranted discussion with reader 2, reader 1 has the opportunity to update their interpretation after considering reader 2’s findings. Reader 1 fills out a second research report (Re-Evaluation Report; see the Supplementary material), captured and stored in our centralized REDCap database, indicating whether the reader would like to make any changes to the initial report. Reader 1 then submits a final clinical report to the patient’s electronic medical record.

#### 3.5.4 Clinical outcomes

As a pragmatic study, biopsy recommendations and other clinical decisions are left to the discretion of the patient’s medical team as per clinical routine. We will review patient medical records to extract relevant outcomes, including whether a biopsy was recommended, whether a biopsy was performed (including technique), and the outcome of any biopsy: number of systematic cores and their locations, number of targeted cores and the corresponding target location, Gleason score and percentage of Gleason patterns per core, ductal or acinar type for any carcinoma, presence of cribriform pattern, presence of intraductal carcinoma, and presence of perineural invasion. If clinical interpretation includes notes of other poor prognostic pathology features, we will also record these [38]. If clinical genomic, pathomic, or other tests are performed on the tumor specimen (eg, Decipher and Artera), we will record these results. If the patient undergoes radical prostatectomy, the final pathology will be recorded, including Gleason score, percentage of Gleason patterns, perineural invasion, tumor features (acinar, ductal, intraductal carcinoma, and cribriform pattern), extraprostatic extension, seminal vesicle invasion, etc.

### 3.6 Study schema for ART-Pro-2

**Fig. 2:**
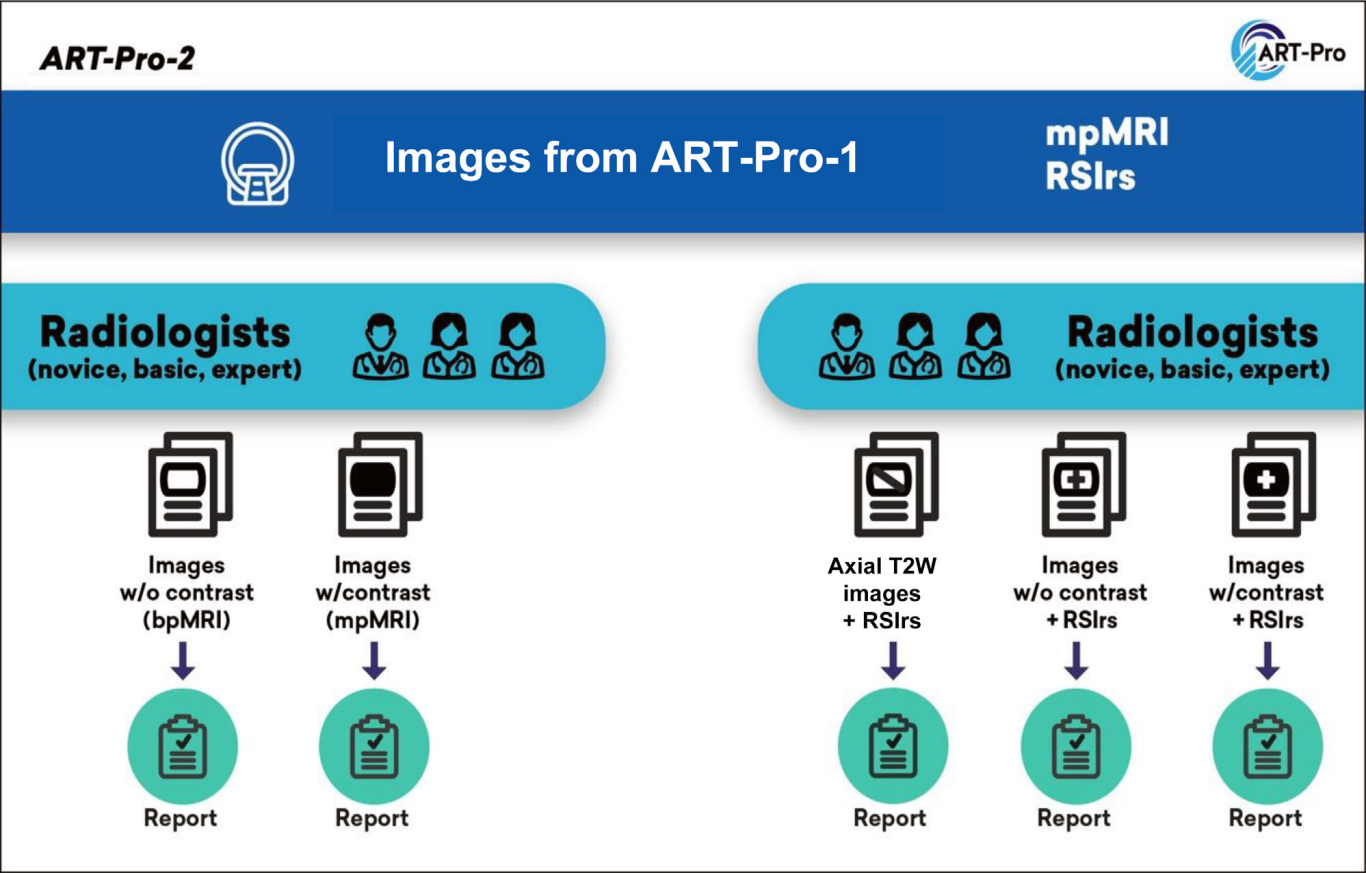
In ART-Pro-2, radiologists of different levels of experience (novice, basic, and expert according to the ESUR/ESUI criteria) evaluate patient images from ART-Pro-1. For patient cases where they are assigned to multiparametric MRI (mpMRI; left), the radiologists provide a report using only biparametric MRI (bpMRI; no contrast) and then a second report after reviewing the dynamic contrast-enhanced images (full mpMRI). For patient cases where they are assigned to Restriction Spectrum Imaging restriction score (RSIrs) plus mpMRI (right), the radiologists provide three reports, in the following order, based only on: (1) axial *T_2_*-weighted (T2W) images and RSIrs maps, (2) bpMRI plus RSIrs maps, and (3) full mpMRI plus RSIrs maps. ESUI = European Association of Urology Section of Urological Imaging; ESUR = European Society of Urogenital Radiologists; MRI = magnetic resonance imaging; w/ = with; w/o = without.

A schematic presentation of ART-Pro-2 is provided in Figure 2.

### 3.7 MRI interpretation in ART-Pro-2

ART-Pro-2 will be conducted retrospectively and will not impact patient care. Radiologists will be categorized by level of experience for prostate MRI based on the joint European Society of Urogenital Radiologists (ESUR) and the EAU Section of Urological Imaging (ESUI) criteria: novice prostate radiologists defined as having read <400 cases, basic prostate radiologists defined as having read ≥400 and <1000 cases, and expert prostate radiologists defined as having read ≥1000 cases [11]. The design of ART-Pro-2 in evaluating nonexpert readers retrospectively allows for “locking” of reader experience level, whereas if evaluated over the ART-Pro-1 trial period, readers could change (by criteria) from being novice to basic and/or expert. Each radiologist will be provided a list of 100 patient cases to review, 50 with mpMRI and 50 with RSIrs plus mpMRI. Case lists will be generated as random permutations of cases while requiring that (1) no radiologist be assigned cases from the institution where he/she works (to ensure that the radiologist has not seen the cases before), (2) each patient case is assigned to at least three radiologists as mpMRI (one from each of the experience levels), and (3) each patient case is assigned to at least three (different) radiologists (one from each of the experience levels) as RSIrs plus mpMRI. Thus, each patient case will be reviewed by six radiologists (two from each of the experience levels). Image sets will be provided as sessions using the MIM Zero Footprint platform (MIM Software, Cleveland, OH, USA) to facilitate individual work lists and presentation of image subsets.

#### 3.7.1 Multiparametric MRI patient cases

For mpMRI patient cases, the radiologist will first be presented with bpMRI images and will complete a REDCap report with own findings according to PI-RADS v2.1. The radiologist will then proceed to review the DCE images for the case and will complete a second REDCap report according to PI-RADS v2.1. In each report, the radiologist will provide an estimate of the probability of csPCa per lesion and per patient.

#### 3.7.2 RSIrs plus mpMRI patient cases

For RSIrs plus mpMRI patient cases, the radiologist will first be presented with only RSIrs maps and axial T2W images. The radiologist will complete a REDCap report based only on images from these two series. As PI-RADS does not apply to RSIrs plus axial T2W images, these reports will use the 5-point Likert scale used in the PROMIS trial: highly unlikely (1), unlikely (2), equivocal (3), likely (4), or highly likely (5). The radiologist will then proceed to review the full bpMRI image set (ie, adding conventional DWI and ADC) and will complete a second REDCap report according to PI-RADS v2.1 guidelines. Finally, the radiologist will review DCE images and complete a third REDCap report for mpMRI according to PI-RADS v2.1. In each report, the radiologist will provide an estimate of the probability of csPCa per lesion and per patient.

## 4 Statistical analysis

We expect bpMRI to be noninferior to mpMRI among expert radiologists for the detection of csPCa and avoidance of unnecessary biopsies. We expect that expert radiologists will have superior performance to nonexperts with bpMRI and with mpMRI. However, we hypothesize that adding RSIrs to bpMRI will facilitate objective MRI interpretation so that nonexpert radiologists using RSIrs plus bpMRI will have noninferior performance to experts using bpMRI or mpMRI. We expect that RSIrs as a stand-alone quantitative biomarker will be noninferior to qualitative mpMRI for discriminating patients with csPCa from those without csPCa.

### 4.1 Analyses for ART-Pro-1

#### 4.1.1 Statistical analysis plan for primary and key secondary objectives

An exploratory data analysis will be conducted, and visualization tools, including the scatterplot, boxplot, and histogram, will be used to examine the data and potential missingness [39]. For all tests below, the significance level of 0.05 will be used in the statistical testing. All analyses will be conducted using software R 4.3.1.

For examining the noninferiority of bpMRI versus mpMRI for the detection of csPCa, we will conduct one-sided noninferiority tests of correlated proportions, to examine the sensitivity and specificity of the following paired readings: reader 1 bpMRI versus reader 1 mpMRI and reader 2 bpMRI versus reader 1 mpMRI. For both paired comparisons, the outcome of each reading for each patient is the binary result of positive or negative MRI. A McNemar's test will be used to test the null hypothesis that the differences in the probabilities (% of second test minus % of the first test) of positive MRI (for testing on sensitivity) and negative MRI (for testing on specificity) from the two readings in the paired comparison are larger than the predetermined noninferiority margins [40]. The *p* values will be reported for all tests.

For examining the noninferiority of RSIrs versus mpMRI, we will compare the areas under the receiver operating characteristic (ROC) curve (AUCs) of using RSIrs (continuous variable) to predict the binary outcome of csPCa versus using mpMRI (ordinal categorical variable; treat like continuous variable) to predict the outcome [41]. A noninferiority test will be conducted to compare the two ROC curves associated with the two predictors at significance level 0.05.

#### 4.1.2 Sample size calculations

All power calculations are conducted using software PASS 14.0.9 and R 4.3.1, unless otherwise indicated.

For testing the sensitivity, we expect the sensitivity to be 90% from the second test in each of the paired comparisons (reader 1 mpMRI), and assume a margin of 8% in defining noninferiority and an actual difference of 0%. We expect the percentage of probability of the first test giving a positive result while the second giving a negative result will be 5%. A sample size of 145 patients achieves at least 80% power at significance level 0.05. We plan to enroll 500 patients and expect that 30% will be diagnosed with csPCa, yielding 175 analyzable patients for the evaluation of sensitivity. Given the design of the trial, missing data are expected to be low (3%). Therefore, with the proposed sample size, we will have ample power in the above statistical tests.

For testing the specificity, we expect the specificity to be 40% from the second test in each of the paired comparisons, and assume a margin of 10% in defining noninferiority [42] and an actual difference of 0%. We expect the percentage of probability of the first test giving a negative result while the second giving a positive result will be 5%. Of 500 patients enrolled, 350 are expected to not be diagnosed with csPCa (either no biopsy due to low clinical/imaging risk or biopsy negative for csPCa), with a missing data rate of 3%. A sample size of 340 analyzable patients achieves at least 80% power at significance level 0.05.

For testing the noninferiority of the ROC curves associated with RSIrs and mpMRI, a preliminary study of 476 patients indicates that the AUC of the model with RSIrs is 0.747 (95% confidence interval [CI]: 0.7025–0.7884), while the AUC of the model with mpMRI is 0.767 (95% CI: 0.726–0.8085). With the proposed sample size, we will have at least 80% power in the noninferiority test with a margin of 8%. The power analysis is conducted with R 4.3.1 and package pROC.

### 4.2 Analyses for ART-Pro-2

Statistical analyses for ART-Pro-2 will be analogous to those described for ART-Pro-1. In ART-Pro-2, each radiologist will give sequential reports on each assigned patient case, that is, either (1) bpMRI and (2) mpMRI or (3) RSIrs plus T2W axial, (4) RSIrs plus bpMRI, and (5) RSIrs plus mpMRI. We will compare each approach (1 and 3–5) to mpMRI in an analogous manner to that in ART-Pro-1 using McNemar’s test and AUCs. These analyses will be performed within each stratum of ESUR radiologist expertise, testing for noninferiority compared with mpMRI PI-RADS for ESUR-defined experts (essentially a validation of ART-Pro-1), radiologists with basic prostate MRI proficiency, and novices. In terms of statistical power, each of these ART-Pro-2 subgroups will have the same statistical power as the primary and key secondary analyses of ART-Pro-1.

Additionally, we will estimate the effect of radiologist expertise for each approach (1–5) in linear mixed-effect models that utilize all the data from ART-Pro-2. Models will take the following form:

csPCastatusscore+experience+(1|patient)+(1|radiologist)

Here, csPCa status is whether the patient was diagnosed with csPCa (binary), score is the PI-RADS or Likert score assigned, and experience is a categorical variable for ESUR/ESUI level of experience. Importance of radiologist experience within each approach (1–5) will be estimated by the parameter estimates for ESUR/ESUI basic proficiency and ESUR/ESUI novice levels, compared with a reference of ESUR/ESUI experts.

### 4.3 Secondary analyses

We will repeat statistical analyses above using an alternate definition of grade group (GG) ≥3 as true positives. GG 1 or benign will continue to be considered true negatives. We will also measure the detection of cancers of each GG (GG 1, 2, 3, or 4–5). GG 1 diagnoses will be considered undesirable overdiagnoses. Rates of overdiagnosis will be compared by McNemar’s test, as above.

The percentage of cases with diagnostic quality imaging as per PI-QUAL (as rated by reader 1 in ART-Pro-1) will be measured for each site and for the study, overall. The percentage of cases with significant distortion of DWI will be measured for each site and for the study, overall. The percentage of such cases where distortion correction was deemed diagnostically helpful will be measured for each site and for the study, overall.

Inter-reader reliability of PI-QUAL and bpMRI PI-RADS scores will be summarized using Cohen’s kappa, comparing ART-Pro-1 reader 1 with ART-Pro-1 reader 2.

We will calculate the percentage of csPCa detected on systemic biopsy only, targeted biopsy only, or both.

Radiologists estimate the probability of csPCa on biopsy for each patient and record this in the REDCap forms. We have previously reported objective estimates of probability of csPCa for RSIrs maximum in the prostate [35]. For ART-Pro patients who undergo biopsy, we will construct ROC curves for radiologists’ and RSIrs estimates, with 95% CIs via bootstrapping. We will compare discriminative performance for expert radiologists, nonexpert radiologists, and RSIrs. We will also visualize calibration for each of these via qq plots [43].

In ART-Pro-2, the overall performance of each approach (1–5) will also be compared by measuring the Akaike information criterion for each model.

## 5 Summary

Prostate mpMRI improves the diagnostic pathway for PCa by increasing the detection of csPCa while also avoiding unnecessary biopsies. The major limitations of mpMRI are the need for IV contrast and its dependence on user expertise. The ART-Pro trial aims to test whether IV contrast can be avoided in the setting of standardized, state-of-the-art image acquisition, with or without addition of a quantitative imaging biomarker, RSIrs. Critically, ART-Pro will study whether novel technology can help nonexpert radiologists achieve performance comparable with expert radiologists, even without the use of IV contrast. In addition, unlike other studies evaluating mpMRI and bpMRI for csPCa detection, ART-Pro employs fully standardized image acquisition protocols across multiple centers. Test characteristics of RSIrs will additionally be evaluated as a standalone, quantitative biomarker. The primary endpoint of ART-Pro is biopsy-confirmed csPCa. A key secondary endpoint is unnecessary biopsies (ie, any biopsy resulting in no cancer or only GG 1).

ART-Pro is being conducted in two linked stages. ART-Pro-1 includes expert readers at five centers of excellence and will yield a carefully curated dataset under ideal conditions. ART-Pro-1 will answer several key questions: (1) Can IV contrast be avoided when expert radiologists are available? (2) Does the use of RSIrs facilitate omission of IV contrast? (3) How does performance of RSIrs alone (as an objective quantitative biomarker) compare with expert bpMRI and mpMRI interpretation in a multicenter study? ART-Pro-2 will leverage the carefully curated dataset from ART-Pro-1 to investigate the impact of IV contrast and RSIrs maps, respectively, on the accuracy of nonexpert radiologists. Since two of the drawbacks to prostate mpMRI (variable interpretation of results and false-positive findings) are exacerbated in nonexpert radiologists, investigating these prostate MRI techniques in nonexperts through ART-Pro-2 will be invaluable for determining the utility of the techniques.

ART-Pro’s pragmatic design facilitates conduct of the study but also has some limitations. Biopsy decisions are made as per clinical routine and are not prescribed by the study, so biopsy may not be performed for some patients despite suspicious MRI. On the contrary, this reflects real-world practice. We expect that conducting the study at centers of excellence will mitigate the risk of nonstandard recommendations, and we have accounted for the possibility of patients declining a recommended biopsy in our power analysis. Clinical decisions for each patient are made based on the final recommendations of one radiologist (ART-Pro-1 reader 1). It is obviously not possible to have the same patient undergo two separate decisions, and our design allows for both reader 1 and reader 2 to influence the biopsy decision and biopsy targets. An alternative approach would be a randomized trial where patients are evaluated with only bpMRI, mpMRI, or RSIrs plus bpMRI. Such a randomized trial would suffer drawbacks of requiring many more patients (because of nonpaired statistical comparisons) and a considerable consenting effort that would inevitably slow accrual and introduce biases because some patient populations are less able to participate in research studies (eg, because of language barriers, complex wording of consent forms, burden of additional visits to complete consent, etc.). ART-Pro-2 has a retrospective design, meaning that the ART-Pro-2 radiologists do not influence biopsy decisions. They may, for example, identify additional lesions that were not targeted on biopsy. Given that each patient can have only one biopsy recommendation and the first biopsy procedure, we believe that the most ethical and most accurate approach is to have two expert radiologists at a center of excellence to influence biopsy recommendations that lead to the gold standard reference for the study’s primary outcome (presence of csPCa on biopsy).

ART-Pro has the potential to make several meaningful impacts on patient care. If bpMRI is proved noninferior to mpMRI for the detection of csPCa in the setting of standardized protocols and technology, patients could benefit from avoidance of discomfort and risks of IV contrast. This would also improve accessibility and decrease the cost of prebiopsy prostate MRI. Validation of RSIrs as a quantitative biomarker could improve objective interpretation and increase reproducibility of results for readers across a range of skill levels. If ART-Pro-2 demonstrates that nonexpert radiologists using only RSIrs and axial T2W series have comparable accuracy to expert radiologists interpreting complete mpMRI, this would mean that it may be possible to adopt a very short prostate MRI examination that could widely be implemented to increase the capacity for prebiopsy MRI significantly, while ensuring reproducible and accurate results. Additionally, the carefully curated dataset created in ART-Pro-1 will be useful for future prostate MRI studies. This dataset could be used, for example, to validate current and future AI-based prostate MRI tools and to measure their performance. Thus, ART-Pro not only will evaluate current protocols and technologies for prostate mpMRI, but also has the potential to improve future prostate MRI research.

We anticipate full accrual by mid-2026, with all participants meeting the primary endpoint by the end of 2026.

  ***Author contributions*:** Tyler M. Seibert had full access to all the data in the study and takes responsibility for the integrity of the data and the accuracy of the data analysis.

  *Study concept and design*: Baxter, Conlin, Bagrodia, Barrett, Bartsch, Brau, Cooperberg, Dale, Guidon, Hahn, Harisinghani, Javier-DesLoges, Kamran (Capuano), Kane, Kuperman, Margolis, Murphy, Nakrour, Ohliger, Rakow-Penner, Shabaik, Simko, Tempany, Wehrli, Woolen, Zou, Seibert.

*Acquisition of data*: Baxter, Conlin, Barrett, Hahn, Harisinghani, Margolis, Murphy, Nakrour, Woolen, Seibert.

*Analysis and interpretation of data*: Conlin, Dale, Seibert.

*Drafting of the manuscript*: Baxter, Conlin, Seibert.

*Critical revision of the manuscript for important intellectual content*: Baxter, Conlin, Bagrodia, Barrett, Bartsch, Brau, Cooperberg, Dale, Guidon, Hahn, Harisinghani, Javier-DesLoges, Kamran (Capuano), Kane, Kuperman, Margolis, Murphy, Nakrour, Ohliger, Rakow-Penner, Shabaik, Simko, Tempany, Wehrli, Woolen, Zou, Seibert.

*Statistical analysis*: Zou, Seibert.

*Obtaining funding*: Seibert.

*Administrative, technical, or material support*: Bartsch, Kuperman.

*Supervision*: Seibert.

*Other*: None.

  ***Financial disclosures:*** Tyler M. Seibert certifies that all conflicts of interest, including specific financial interests and relationships and affiliations relevant to the subject matter or materials discussed in the manuscript (eg, employment/affiliation, grants or funding, consultancies, honoraria, stock ownership or options, expert testimony, royalties, or patents filed, received, or pending), are the following: Anders M. Dale is a founder of and holds equity in CorTechs Labs, Inc, and serves on its scientific advisory board; he is also a member of the scientific advisory board of Human Longevity, Inc, and receives funding through research agreements with GE Healthcare. Michael E. Hahn reports honoraria from Multimodal Imaging Services Corporation and research funding from GE Healthcare. Rebecca Rakow-Penner has an equity interest in CorTechs Labs and CureMetrix, serves on the scientific advisory board of Imagine Scientific, and has received consulting fees from Bayer and research funding from GE Healthcare. Arnaud Guidon and Aditya Bagrodia are employees of GE Healthcare. Tyler M. Seibert reports honoraria from CorTechs Labs, Varian Medical Systems, WebMD, GE Healthcare, Multimodal Imaging Services, and Janssen; he has an equity interest in CorTechs Labs, Inc, and serves on its scientific advisory board; he has also received in-kind research support from GE Healthcare via a research agreement with the University of California, San Diego. These companies might potentially benefit from the research results. The terms of these arrangements have been reviewed and approved by the University of California, San Diego in accordance with its conflict-of-interest policies.

  ***Funding/Support and role of the sponsor*:** This work was supported by GE Healthcare.

  ***Acknowledgments*:** The PI presented the study to patient support groups and at a public patient support group during study development. We would like to acknowledge two patient representatives, Tony Collier and Richard Taylor, who were engaged in the study design.
