# Supplementary material for: Advanced Restriction Imaging and Reconstruction Technology for Prostate Magnetic Resonance Imaging (ART-Pro): A Study Protocol for a Multicenter, Multinational Trial Evaluating Biparametric Magnetic Resonance Imaging and Advanced, Quantitative Diffusion Magnetic Resonance Imaging for the Detection of Prostate Cancer
Source: Eur Urol Open Sci. 2024 Dec 20;71:132–43. doi: 10.1016/j.euros.2024.12.003 (PMC11730575; doi:10.1016/j.euros.2024.12.003)
Supplement: Supplementary Data 1 [file mmc1.pdf]

Cambridge University Hospitals NHS Foundation Trust

VOLUNTEER CONSENT FORM

Volunteer Identification Number for this trial:

Title of Project: **Development of novel magnetic resonance techniques**

Name of Lead Investigator: **Dr Martin Graves**

Please initial box

1. I confirm that I have read and understand the information sheet version 4.0 dated 18 Feb 2020 for the above study and have had the opportunity to consider the information, ask questions and have had these answered satisfactorily. ☐

2. I understand that my participation is voluntary and that I am free to withdraw at any time, without giving any reason, without my medical care or legal rights being affected. ☐

3. I understand that images from the MR examinations and sections of any of my medical records (where they are relevant to my taking part in this study) may be looked at by members of the research team, and also made available to the Trust Research & Development Department for audit and monitoring purposes. I understand that such access will be in accordance with the General Data Protection Regulation, and medical confidentiality rules. I give permission for these individuals to have access to my records. ☐

4. I understand that this research scan is not a clinical scan and I will not be explicitly investigated for any medical disorders or diagnostic purposes; however if a significant finding is noticed, I agree to be informed of the result. ☐

5. I understand that the data from this study, suitably anonymised, may be used in scientific publications and presentations. ☐

6. I agree that the data obtained may be shared in anonymous form through local, national or international data repositories. ☐

7. I give permission for a report of any unexpected abnormalities found during my MRI examination to be communicated to my General Practitioner. ☐

8. I agree to take part in the above study. ☐

Name of Research Subject<br>*(Please print)*	Date	Signature
Name of Research Team member<br>*(Please print)*	Date	Signature

2 copies required: top copy for researcher; one copy for volunteer

Cambridge University Hospitals NHS Foundation Trust

Optional – please insert GP details below:

Name of GP: ____

Address: ____

____

____

____
