# Supplementary material for: Advanced Restriction Imaging and Reconstruction Technology for Prostate Magnetic Resonance Imaging (ART-Pro): A Study Protocol for a Multicenter, Multinational Trial Evaluating Biparametric Magnetic Resonance Imaging and Advanced, Quantitative Diffusion Magnetic Resonance Imaging for the Detection of Prostate Cancer
Source: Eur Urol Open Sci. 2024 Dec 20;71:132–43. doi: 10.1016/j.euros.2024.12.003 (PMC11730575; doi:10.1016/j.euros.2024.12.003)
Supplement: Supplementary Data 4 [file mmc4.pdf]

Re-Evaluation Form - Reader 1 (SOC)

A A A

Please complete the form below.

Please note if you will make any changes to your report after seeing the report from the second reader.

Patient ID
Input pre-assigned Patient ID only. DO NOT include PHI.
* must provide value

Radiologist Last Name
* must provide value

Would You Like to Change Any of Your MRI Findings? *must provide value

- No
- Yes - Adjust a Lesion Score
- Yes - Add a Lesion
- Yes - Exclude a Lesion

reset

If Yes, Please Include Details:

Expand

Submit

Powered by REDCap
