# Supplementary material for: Advanced Restriction Imaging and Reconstruction Technology for Prostate Magnetic Resonance Imaging (ART-Pro): A Study Protocol for a Multicenter, Multinational Trial Evaluating Biparametric Magnetic Resonance Imaging and Advanced, Quantitative Diffusion Magnetic Resonance Imaging for the Detection of Prostate Cancer
Source: Eur Urol Open Sci. 2024 Dec 20;71:132–43. doi: 10.1016/j.euros.2024.12.003 (PMC11730575; doi:10.1016/j.euros.2024.12.003)
Supplement: Supplementary Data 5 [file mmc5.docx]

**Supplementary material**

**Protocol design**

For axial scans, participating institutions used a mixture of 16 cm and 20 cm FOV sizes. To select between these options, a volunteer was scanned using both FOV sizes (maintaining constant in-plane resolution in accordance with PI-RADS v2.1), and both sets of images were distributed to all participating radiologists for review. By majority vote, the 16 cm FOV was selected for axial imaging.

Axial *T_2_*-weighted images were also obtained from the same volunteer using two different acquisition strategies, cartesian fast spin echo (FSE) and radial “Periodically Rotated Overlapping ParallEL Lines with Enhanced Reconstruction” (PROPELLER) and compared by all participating radiologists. Cartesian FSE was selected by majority vote for inclusion in the protocol.

Imaging parameters for the other *T_2_*-weighted, *T_1_*-weighted, conventional DWI, and dynamic contrast-enhanced scans were determined via discussion among all participating radiologists and votes for consensus.

For RSI, resolution and scan coverage were selected for compliance with PI-RADS v2.1. *b*-values were determined from an estimation theory analysis described previously ^1^.

**References**

1. Conlin CC, Seibert TM, Dale AM. Optimal protocol design for diffusion-weighted imaging of the prostate: an estimation theory examination of parameter estimate variance. Published online February 28, 2022. doi:10.1101/2022.02.26.22271561
