# Supplementary material for: Advanced Restriction Imaging and Reconstruction Technology for Prostate Magnetic Resonance Imaging (ART-Pro): A Study Protocol for a Multicenter, Multinational Trial Evaluating Biparametric Magnetic Resonance Imaging and Advanced, Quantitative Diffusion Magnetic Resonance Imaging for the Detection of Prostate Cancer
Source: Eur Urol Open Sci. 2024 Dec 20;71:132–43. doi: 10.1016/j.euros.2024.12.003 (PMC11730575; doi:10.1016/j.euros.2024.12.003)
Supplement: Supplementary Data 2 [file mmc2.pdf]

Reader 1 (Standard of Care) Report

AAA

Please complete the form below.

Patient ID

Input pre-assigned Patient ID only. DO NOT include PHI.

* must provide value

Radiologist Last Name

* must provide value

Patient History

Patient History Known at Time of Read

* must provide value

Expand

Include History Known at Time of Read, e.g., PSA Values, Prior MRI Findings, Prior Biopsy, Prior Treatment, etc.

Patient Category

* must provide value

- Active Surveillance
- Biopsy Naïve
- Prior Negative Biopsy
- Unknown

reset

General Findings

Prostate Volume (cc):

* must provide value

Hardware/Implant Present: * must provide value

- None
- Hip Instrumentation
- Urolift
- Fiducials
- Spacer
- Other

Other:

T1W: Hemorrhage or Other Notable Finding? * must provide value

- Yes
- No

reset

Other T1W Notes:

Previous MRI Visible for Comparison at Time of Read? * must provide value

- Yes - PI-RADS Unknown
- Yes - PI-RADS 1-2
- Yes - PI-RADS 3-5
- No

reset

Previous (Most Recent) MRI Date:

Previous MRI Notes/Results:

Expand

PI-QUAL Evaluation

Is T2-WI of Diagnostic Quality? * must provide value

- Yes
- No

reset

Is DWI of Diagnostic Quality? * must provide value

- Yes
- No

reset

Other PI-QUAL Notes:

Expand

Lesion Information

Fill out information for all applicable lesions. If < 4 lesions, leave non-applicable lesion fields blank.

Add Lesion?

* must provide value

- (•) Yes
- No

reset

Lesion 1

Lesion Location

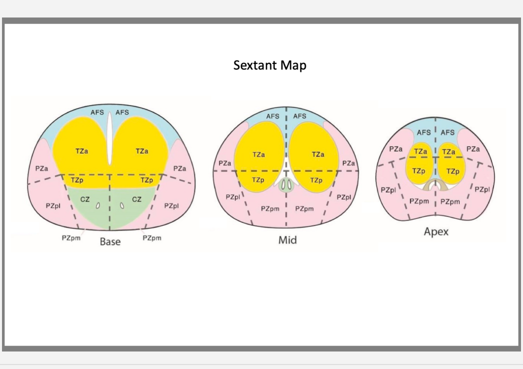

Base		Mid		Apex	
Right	Left	Right	Left	Right	Left
AFS	AFS	AFS	AFS	AFS	AFS
TZa	TZa	TZa	TZa	TZa	TZa
TZp	TZp	TZp	TZp	TZp	TZp
PZa	PZa	PZa	PZa	PZa	PZa
PZpl	PZpl	PZpl	PZpl	PZpl	PZpl
PZpm	PZpm	PZpm	PZpm	PZpm	PZpm
CZ	CZ				

Other Lesion Location Notes:

Expand

Size

Lesion Diameter (cm)	Other Lesion Size Notes
**Largest Diameter**	
**Second Largest Diameter**	
**Craniocaudal Diameter**	Expand

Please also annotate diameters as key images or screenshots in PACS.

DWI Evaluation

DWI Score	Mean ADC	Other DWI Notes
1 2 3 4 5 X reset		Expand

T2W Evaluation

T2W Score	Other T2W Notes
1 2 3 4 5 reset	Expand

PI-RADS Evaluation (bpMRI)

PI-RADS Score	Other PI-RADS Notes
1 2 3 4 5 reset	Expand

Other

EPE Findings	Other EPE Notes
No capsular contact<br>Minimal capsular contact<br>Broad capsular contact<br>Broad capsular contact w/ bulging<br>Gross EPE<br>reset	Expand

**What is the percentage probability that this patient would have neurovascular bundle (NVB) involved on prostatectomy?**	
**What is the percentage probability that this patient would have seminal vesicle invasion (SVI) on prostatectomy?**	

% Probability of GS ≥ 7 Cancer in Lesion:

Add Lesion?

- (•) Yes

reset

Lesion 2

Lesion Location

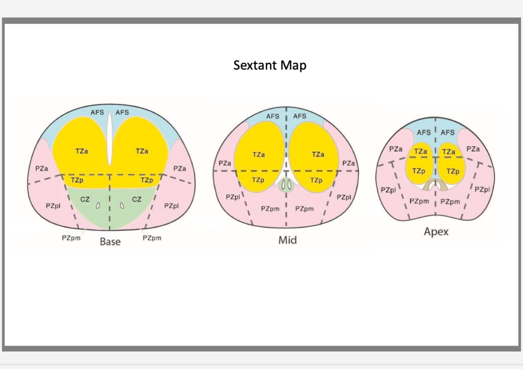

Base		Mid		Apex	
Right	Left	Right	Left	Right	Left
AFS	AFS	AFS	AFS	AFS	AFS
TZa	TZa	TZa	TZa	TZa	TZa
TZp	TZp	TZp	TZp	TZp	TZp
PZa	PZa	PZa	PZa	PZa	PZa
PZpl	PZpl	PZpl	PZpl	PZpl	PZpl
PZpm	PZpm	PZpm	PZpm	PZpm	PZpm
CZ	CZ				

Other Lesion Location Notes:

Expand

Size

Lesion Diameter (cm)	Other Lesion Size Notes
**Largest Diameter**	
**Second Largest Diameter**	
**Craniocaudal Diameter**	Expand

Please also annotate diameters as key images or screenshots in PACS.

DWI Evaluation

DWI Score	Mean ADC	Other DWI Notes
1 2 3 4 5 X reset		Expand

T2W Evaluation

T2W Score	Other T2W Notes
1 2 3 4 5 reset	Expand

PI-RADS Evaluation (bpMRI)

PI-RADS Score	Other PI-RADS Notes
1 2 3 4 5 reset	Expand

Other

EPE Findings	Other EPE Notes
No capsular contact<br>Minimal capsular contact<br>Broad capsular contact<br>Broad capsular contact w/ bulging<br>Gross EPE<br>reset	Expand

**What is the percentage probability that this patient would have neurovascular bundle (NVB) involved on prostatectomy?**	
**What is the percentage probability that this patient would have seminal vesicle invasion (SVI) on prostatectomy?**	

% Probability of GS ≥ 7 Cancer in Lesion:

Add Lesion?

- (•) Yes

reset

Lesion 3

Lesion Location

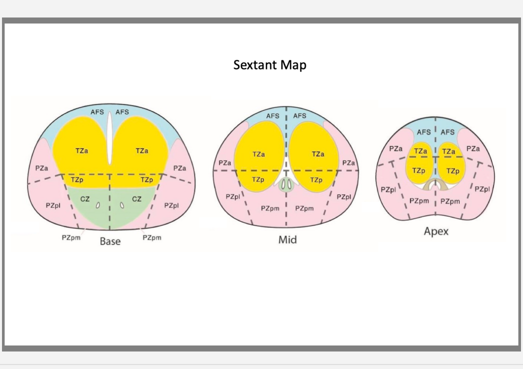

Base		Mid		Apex	
Right	Left	Right	Left	Right	Left
AFS	AFS	AFS	AFS	AFS	AFS
TZa	TZa	TZa	TZa	TZa	TZa
TZp	TZp	TZp	TZp	TZp	TZp
PZa	PZa	PZa	PZa	PZa	PZa
PZpl	PZpl	PZpl	PZpl	PZpl	PZpl
PZpm	PZpm	PZpm	PZpm	PZpm	PZpm
CZ	CZ				

Other Lesion Location Notes:

Expand

Size

Lesion Diameter (cm)	Other Lesion Size Notes
**Largest Diameter**	
**Second Largest Diameter**	
**Craniocaudal Diameter**	Expand

Please also annotate diameters as key images or screenshots in PACS.

DWI Evaluation

DWI Score	Mean ADC	Other DWI Notes
1 2 3 4 5 X reset		Expand

T2W Evaluation

T2W Score	Other T2W Notes
1 2 3 4 5 reset	Expand

PI-RADS Evaluation (bpMRI)

PI-RADS Score	Other PI-RADS Notes
1 2 3 4 5 reset	Expand

Other

EPE Findings	Other EPE Notes
No capsular contact<br>Minimal capsular contact<br>Broad capsular contact<br>Broad capsular contact w/ bulging<br>Gross EPE<br>reset	Expand

**What is the percentage probability that this patient would have neurovascular bundle (NVB) involved on prostatectomy?**	
**What is the percentage probability that this patient would have seminal vesicle invasion (SVI) on prostatectomy?**	

% Probability of GS ≥ 7 Cancer in Lesion:

Add Lesion?

- (•) Yes

reset

Lesion 4

Lesion Location

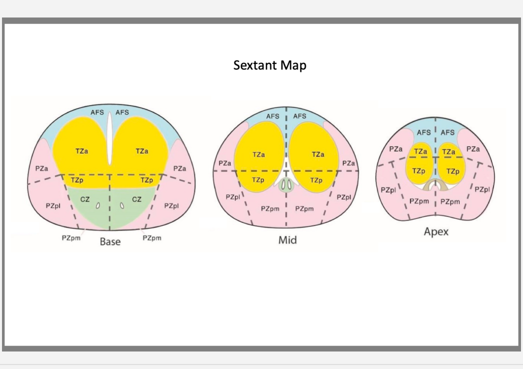

Base		Mid		Apex	
Right	Left	Right	Left	Right	Left
AFS	AFS	AFS	AFS	AFS	AFS
TZa	TZa	TZa	TZa	TZa	TZa
TZp	TZp	TZp	TZp	TZp	TZp
PZa	PZa	PZa	PZa	PZa	PZa
PZpl	PZpl	PZpl	PZpl	PZpl	PZpl
PZpm	PZpm	PZpm	PZpm	PZpm	PZpm
CZ	CZ				

Other Lesion Location Notes:

Expand

Size

Lesion Diameter (cm)	Other Lesion Size Notes
**Largest Diameter**	
**Second Largest Diameter**	
**Craniocaudal Diameter**	Expand

Please also annotate diameters as key images or screenshots in PACS.

DWI Evaluation

DWI Score	Mean ADC	Other DWI Notes
1 2 3 4 5 X reset		Expand

T2W Evaluation

T2W Score	Other T2W Notes
1 2 3 4 5 reset	Expand

PI-RADS Evaluation (bpMRI)

PI-RADS Score	Other PI-RADS Notes
1 2 3 4 5 reset	Expand

Other

EPE Findings	Other EPE Notes
No capsular contact<br>Minimal capsular contact<br>Broad capsular contact<br>Broad capsular contact w/ bulging<br>Gross EPE<br>reset	Expand

**What is the percentage probability that this patient would have neurovascular bundle (NVB) involved on prostatectomy?**	
**What is the percentage probability that this patient would have seminal vesicle invasion (SVI) on prostatectomy?**	

% Probability of GS ≥ 7 Cancer in Lesion:

Gleason Score ≥ 7 Estimation (Overall Anywhere in the Prostate)

After evaluating the images and taking into account everything about the patient, what would you estimate the probability of GS ≥ 7 if the patient underwent a systematic and targeted biopsy?

* must provide value

If you are ready to move onto the mpMRI Report, please go to the next page.

Note: Once you go to the next page, you will not be able to return to this page to edit bpMRI findings.

Next Page >>

Powered by REDCap

Reader 1 (Standard of Care) Report

A A A

mpMRI Read

PI-QUAL Evaluation

Is DCE of Diagnostic Quality? * must provide value

- ○ Yes
- ○ No

reset

Other PI-QUAL DCE Notes:

Expand

Lesion Information

Fill out information for all lesions found with bpMRI. If new lesion found with mpMRI, add new lesion information at bottom of page.

Lesion 1

Lesion Location

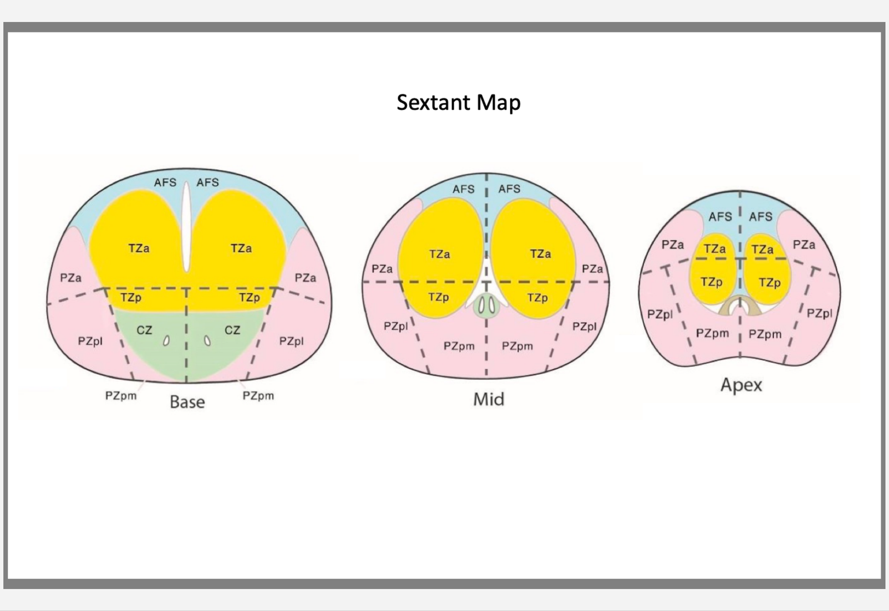

Base		Mid		Apex	
Right	Left	Right	Left	Right	Left
____	____	____	____	____	____

Other Lesion Location Notes:

Expand

bpMRI PI-RADS Score: ____

DCE Evaluation

DCE Score	Other DCE Notes
○ - ○ + ○ Equivocal ○ N/A reset	Expand

PI-RADS Evaluation (mpMRI)

PI-RADS Score	Other PI-RADS Notes
○ 1 ○ 2 ○ 3 ○ 4 ○ 5 reset	Expand

Other

% Probability of GS ≥ 7 Cancer in Lesion:

Lesion 2

Lesion Location

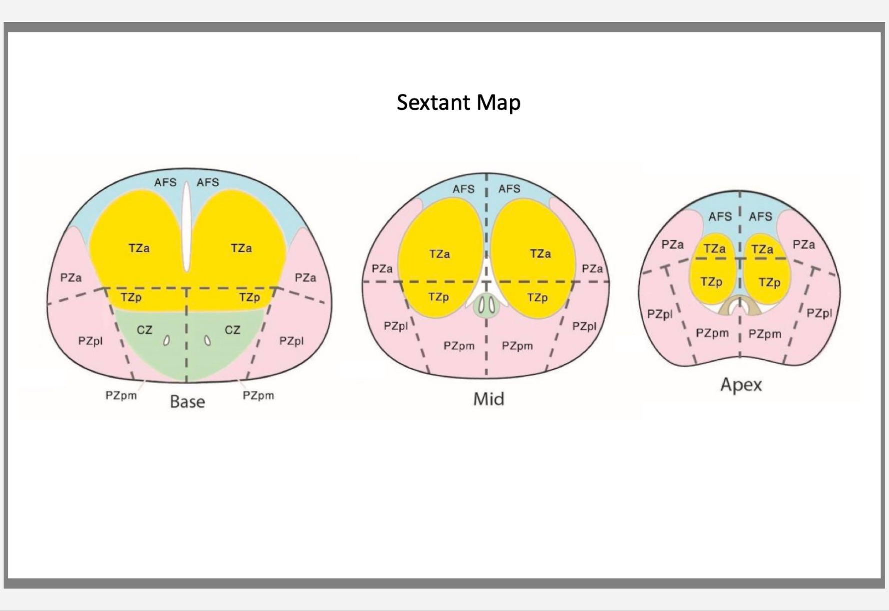

Base		Mid		Apex	
Right	Left	Right	Left	Right	Left
____	____	____	____	____	____

Other Lesion Location Notes:

Expand

bpMRI PI-RADS Score: ____

DCE Evaluation

DCE Score	Other DCE Notes
○ - ○ + ○ Equivocal ○ N/A reset	Expand

PI-RADS Evaluation (mpMRI)

PI-RADS Score	Other PI-RADS Notes
○ 1 ○ 2 ○ 3 ○ 4 ○ 5 reset	Expand

Other

% Probability of GS ≥ 7 Cancer in Lesion:

Lesion 3

Lesion Location

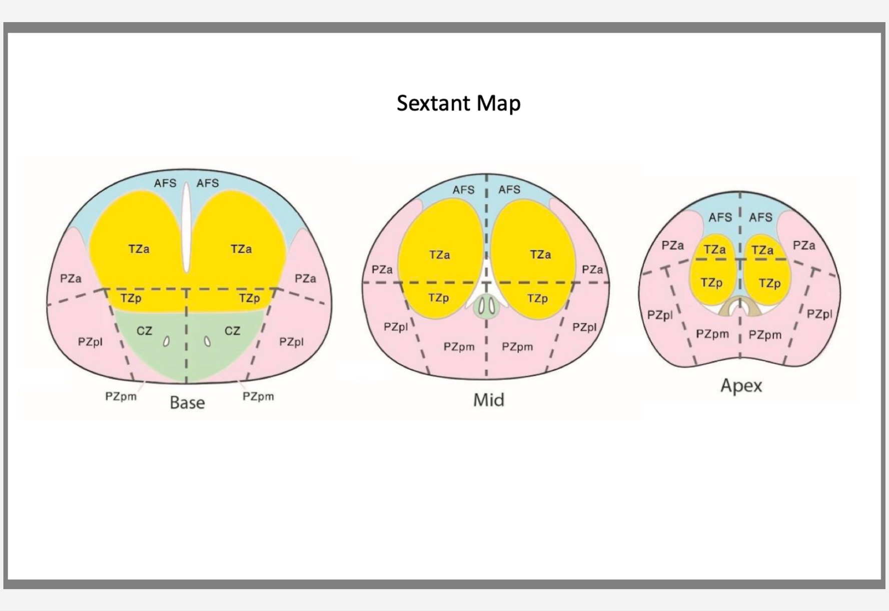

Base		Mid		Apex	
Right	Left	Right	Left	Right	Left
____	____	____	____	____	____

Other Lesion Location Notes:

Expand

bpMRI PI-RADS Score: ____

DCE Evaluation

DCE Score	Other DCE Notes
○ - ○ + ○ Equivocal ○ N/A reset	Expand

PI-RADS Evaluation (mpMRI)

PI-RADS Score	Other PI-RADS Notes
○ 1 ○ 2 ○ 3 ○ 4 ○ 5 reset	Expand

Other

% Probability of GS ≥ 7 Cancer in Lesion:

Lesion 4

Lesion Location

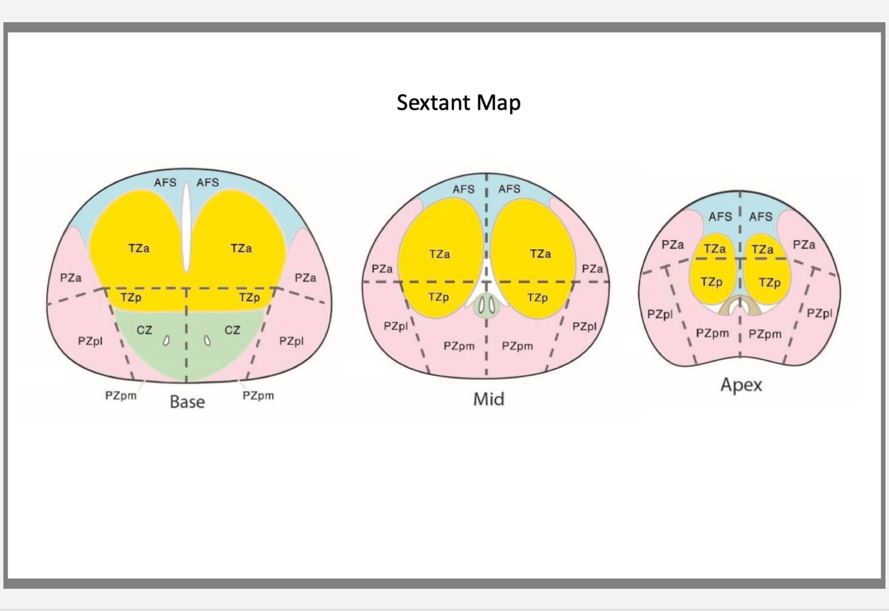

Base		Mid		Apex	
Right	Left	Right	Left	Right	Left
____	____	____	____	____	____

Other Lesion Location Notes:

Expand

bpMRI PI-RADS Score: ____

DCE Evaluation

DCE Score	Other DCE Notes
○ - ○ + ○ Equivocal ○ N/A reset	Expand

PI-RADS Evaluation (mpMRI)

PI-RADS Score	Other PI-RADS Notes
○ 1 ○ 2 ○ 3 ○ 4 ○ 5 reset	Expand

Other

% Probability of GS ≥ 7 Cancer in Lesion:

Add New Lesion (Not Seem with bpMRI)?

- ◉ Yes

reset

New Lesion

Lesion Location

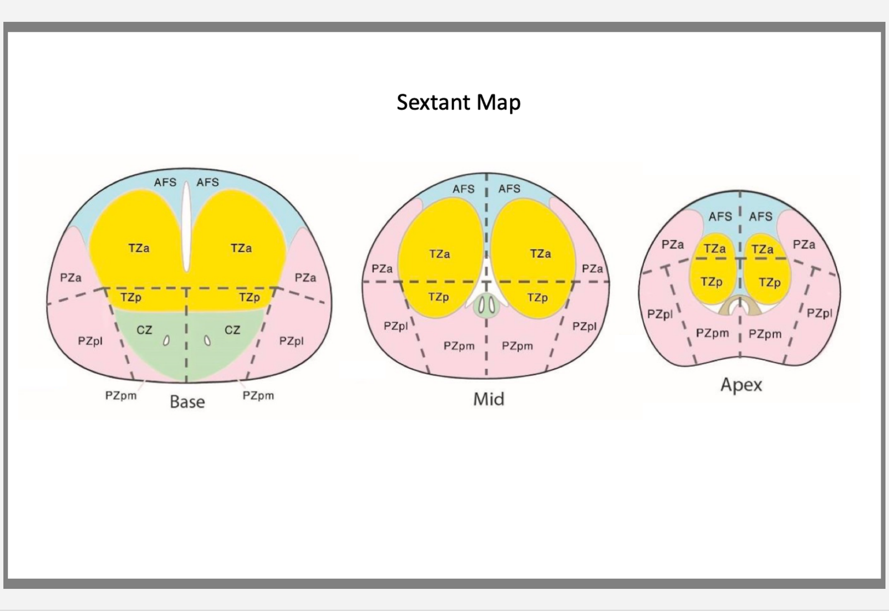

Base		Mid		Apex	
Right	Left	Right	Left	Right	Left
☐ AFS<br>☐ TZa<br>☐ TZp<br>☐ PZa<br>☐ PZpl<br>☐ PZpm<br>☐ CZ	☐ AFS<br>☐ TZa<br>☐ TZp<br>☐ PZa<br>☐ PZpl<br>☐ PZpm<br>☐ CZ	☐ AFS<br>☐ TZa<br>☐ TZp<br>☐ PZa<br>☐ PZpl<br>☐ PZpm	☐ AFS<br>☐ TZa<br>☐ TZp<br>☐ PZa<br>☐ PZpl<br>☐ PZpm	☐ AFS<br>☐ TZa<br>☐ TZp<br>☐ PZa<br>☐ PZpl<br>☐ PZpm	☐ AFS<br>☐ TZa<br>☐ TZp<br>☐ PZa<br>☐ PZpl<br>☐ PZpm

Other Lesion Location Notes:

Expand

Size

Lesion Diameter (mm)	Other Lesion Size Notes
**Largest Diameter** ____<br>**Second Largest Diameter** ____<br>**Craniocaudal Diameter** ____	Expand

Please also annotate diameters as key images or screenshots in PACS.

DWI Evaluation

DWI Score	Mean ADC	Other DWI Notes
○ 1 ○ 2 ○ 3 ○ 4 ○ 5 ○ X reset		Expand

T2W Evaluation

T2W Score	Other T2W Notes
○ 1 ○ 2 ○ 3 ○ 4 ○ 5 reset	Expand

DCE Evaluation

DCE Score	Other DCE Notes
○ - ○ + ○ Equivocal ○ N/A reset	Expand

PI-RADS Evaluation (mpMRI)

PI-RADS Score	Other PI-RADS Notes
○ 1 ○ 2 ○ 3 ○ 4 ○ 5 reset	Expand

Other

EPE Findings	Other EPE Notes
○ No capsular contact<br>○ Minimal capsular contact<br>○ Broad capsular contact<br>○ Broad capsular contact w/ bulging<br>○ Gross EPE<br>reset	Expand

**What is the percentage probability that this patient would have neurovascular bundle (NVB) involved on prostatectomy?**	
**What is the percentage probability that this patient would have seminal vesicle invasion (SVI) on prostatectomy?**	

% Probability of GS ≥ 7 Cancer in Lesion:

Submit

Powered by REDCap
